# Separating Sisters: Shugoshin Protects SA2 at Centromeres but Not at Chromosome Arms

**DOI:** 10.1371/journal.pbio.0030098

**Published:** 2005-03-01

DNA replication leaves the cell with two identical copies of each chromosome. To ensure their proper segregation during the anaphase stage of mitosis, the members of each pair, called sister chromatids, are held together by a protein complex, aptly named cohesin, that links the two not only at the centromere, but also along the chromatid arms. Anaphase is triggered when cohesin is cleaved, by the equally well-named separase. But cleavage is not the only way to remove cohesin from the chromosome; indeed, in humans and other higher eukaryotes, mitotic kinases such as Plk1 remove the majority of cohesin from chromosome arms—but not from the centromere—during prophase and prometaphase.

These facts raise two questions: what is the precise target of Plk1, and what protects centromeric cohesin from removal by the same pathway? Both questions are addressed in a new article in *PLoS Biology*. Jan-Michael Peters and colleagues show that phosphorylation of the cohesin subunit SA2, presumably by Plk1, is required for cohesin removal from chromosome arms in early mitosis, while data from Kim Nasmyth and colleagues suggest that a protein called shugoshin protects centromeric SA2 from such phosphorylation.[Fig pbio-0030098-g001]

**Figure pbio-0030098-g001:**
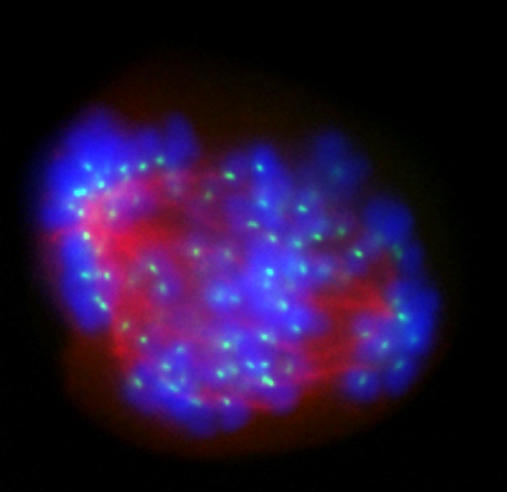
Premature loss of sister chromatid cohesion

Cohesin is composed of multiple subunits, each of which can be phosphorylated at multiple threonine or serine amino acid residues. These subunits include Scc1 (the target of separase), Smc1, and Smc3, plus Scc3 in yeast, and SA1 or SA2 in humans and other higher eukaryotes. By isolating and analyzing cohesin subunits from cells undergoing mitosis, Peters and colleagues deduced that Scc1, SA1, and SA2 are phosphorylated only during mitosis, suggesting that phosphorylation of one or more of them triggers the breakup of cohesin. Further analysis by mass spectrometry allowed them to identify the exact amino acids that bore the phosphates on each subunit. In Scc1, these were clustered around the known sites of separase cleavage. The researchers showed that phosphorylation at these sites is required for efficient cleavage by the enzyme during anaphase, but is not required to dislodge cohesin specifically from the chromosome arms, as this proceeded essentially normally even after these sites were mutated to prevent their phosphorylation.

The same mutation strategy applied to SA2, on the other hand, revealed that phosphorylation of this subunit is essential for dissociating cohesin from the chromosome arms during prometaphase. Interestingly, the mutations did not prevent the ultimate separation of the chromatids at anaphase. This suggests that separase, once it is activated, can cleave cohesin on the arms as well as at the centromere.

Cohesin at the centromere is removed later in mitosis than cohesin bound to chromatid arms, namely, at the metaphase-to-anaphase transition, suggesting centromeric cohesin is protected by a centromere-specific molecule. Possible candidates would be members of the shugoshin family, which are known to prevent unloading of centromeric cohesin during the first division of meiosis, thus keeping chromatids together as homologous chromosomes are separated.

To investigate human shugoshin's mitotic role, Nasmyth and colleagues depleted shugoshin by RNAi. The result was loss of cohesin not only from the arms but also from the centromere, early separation of chromatids, and failure of anaphase, suggesting that shugoshin protects centromeric cohesin. But how? To find out, the authors examined the effect of shugoshin depletion in cells whose SA2 had been mutated to prevent phosphorylation. Strikingly, these cells underwent mitosis successfully. Together, these results suggest that shugoshin's normal mitotic role is to protect centromeric SA2 from phosphorylation, delaying chromatid separation until the moment when the chromosomes are ready to separate, at which time cohesin is cleaved by separase.

The picture that emerges from these two studies is that sister chromatid cohesion is safeguarded throughout early mitosis by shugoshin, which protects centromeric cohesin from the threat of protein kinases that, in the authors' vivid language, “maraud mitotic chromosomes and threaten to destroy their integrity.” This delicate balance of power between kinases and shugoshin means that any upset in the balance may prevent a cell from dividing properly, which often means not dividing at all. (Also see the Primer “Chromosome Cohesion: A Cycle of Holding Together and Falling Apart” [DOI: 10.1371/journal.pbio.0030094].)

